# Type 2 Diabetes Management Among Older Black American Men During the COVID-19 Pandemic: A Qualitative Investigation

**DOI:** 10.1093/geroni/igab046.2727

**Published:** 2021-12-17

**Authors:** Ledric Sherman, DeLawnia Comer-HaGans, Sara Vera

**Affiliations:** 1 Texas A&M University, College Station, Texas, United States; 2 Texas Department of State Health Services, Austin, Texas, United States

## Abstract

As COVID-19 swept across the globe in 2020, it appeared to have infected and killed Black Americans at a disproportionately higher rate. However, few studies have focused specifically on the complications of managing diabetes, expressly type 2 diabetes (T2D), among Black men during the global pandemic. Therefore, the purpose of this qualitative study was to seek understanding of the experiences in managing T2D among Black men during the COVID-19 pandemic. One on one interviews were conducted via Zoom video conferencing with twenty-two (n=22) Black men regarding their experience of managing type 2 diabetes (T2D) in a pandemic environment. Four main themes emerged from the study, which are: (1) stress levels during the pandemic, (2) barriers to managing my diabetes, (3) who/what is helping the most, and (4) communication with health-care provider. As more information becomes available, it is apparent that having diabetes increases the risk for detrimental COVID-19 outcomes (i.e. increased lengths of hospital stays, the need for mechanical ventilation, and mortality. Future research efforts are crucially needed to provide an improved understanding of how individuals across all racial groups are managing diabetes during the COVID-19 pandemic.

